# Cat Anatomist: Exteriorization of a Presumed Digital Nerve Fascicle Following a Cat Scratch Injury

**DOI:** 10.7759/cureus.116018

**Published:** 2026-09-09

**Authors:** Jeremy Jones, Samuel Sternberg, Greg Jacobs

**Affiliations:** 1 Medicine, Alabama College of Osteopathic Medicine, Dothan, USA; 2 College of Science and Mathematics, Auburn University, Auburn, USA; 3 Clinical Simulation Medicine, Alabama College of Osteopathic Medicine, Dothan, USA

**Keywords:** case report, cat scratch, digital nerve, hand trauma, nerve fascicle, peripheral nerve injury

## Abstract

Peripheral nerve injuries of the hand are common; however, exteriorization of a nerve fascicle following minor trauma is exceedingly rare. We present the case of an 80-year-old female who sustained a cat scratch to the dorsum of the hand, resulting in extrusion of a threadlike structure later confirmed to be neural tissue. Initial management included conservative wound care and submission of the specimen as a presumed foreign body. Due to limited clinical information, the specimen underwent gross examination only and was nearly discarded. Following direct communication with pathology, frozen section analysis confirmed the presence of a nerve fascicle. This case highlights the importance of anatomical recognition, accurate specimen labeling, and interdisciplinary communication. It also demonstrates a unique mechanism of peripheral nerve injury involving traction and exteriorization along the presumed course of a dorsal digital nerve.

## Introduction

Peripheral nerve injuries of the hand are frequently encountered in clinical practice and can result from blunt, penetrating, or iatrogenic trauma. These injuries range in severity from neuropraxia to complete transection and may lead to persistent sensory deficits if not properly identified and managed [1-3].

While superficial nerves of the hand are vulnerable to injury due to their anatomical location, the extrusion of a nerve fascicle through the skin following low-energy trauma is highly unusual. Recognition of such structures is critical, as misidentification may result in improper management or missed diagnostic opportunities.

In addition to anatomical knowledge, effective communication with pathology services is essential to ensure appropriate specimen processing. We present a rare case of a presumed digital nerve fascicle exteriorized following a cat scratch injury and discuss the clinical and diagnostic implications.

## Case presentation

An 80-year-old female presented to the emergency department a short time after being scratched by her cat while attempting to pick it up. Her vital signs were unremarkable.

**Figure 1:**
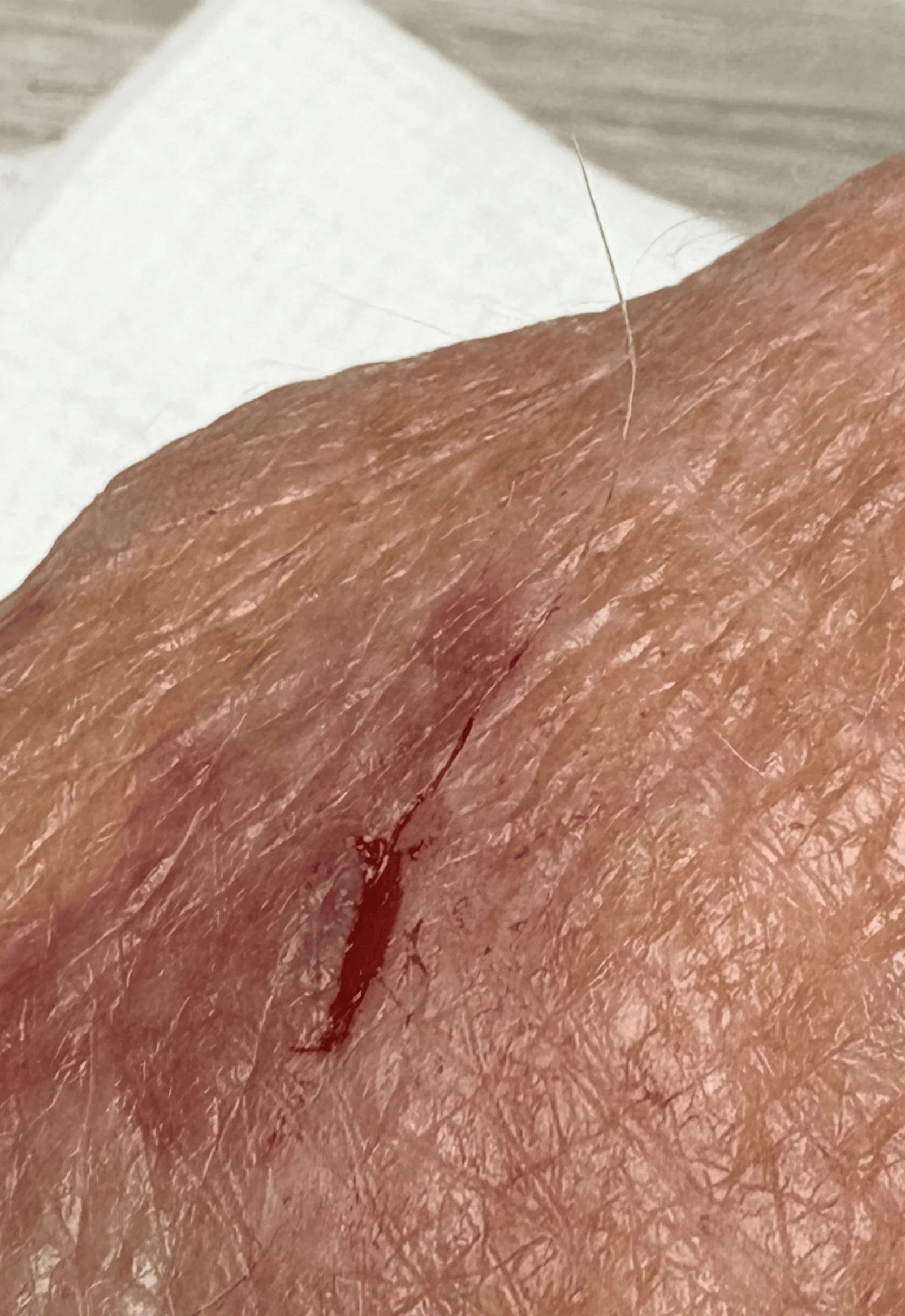
The patient’s wound upon arrival in triage, with a notably long segment of protruding tissue.

Physical examination revealed a linear, narrow, approximately 1 cm laceration on the dorsal aspect of the left hand, of undocumented depth, just proximal to the second metacarpophalangeal joint (Figure 1). A 3-4 cm threadlike structure was protruding from the wound. The patient reported that she and her daughter had attempted to remove the structure but were unable to do so due to pain.

**Figure 2:**
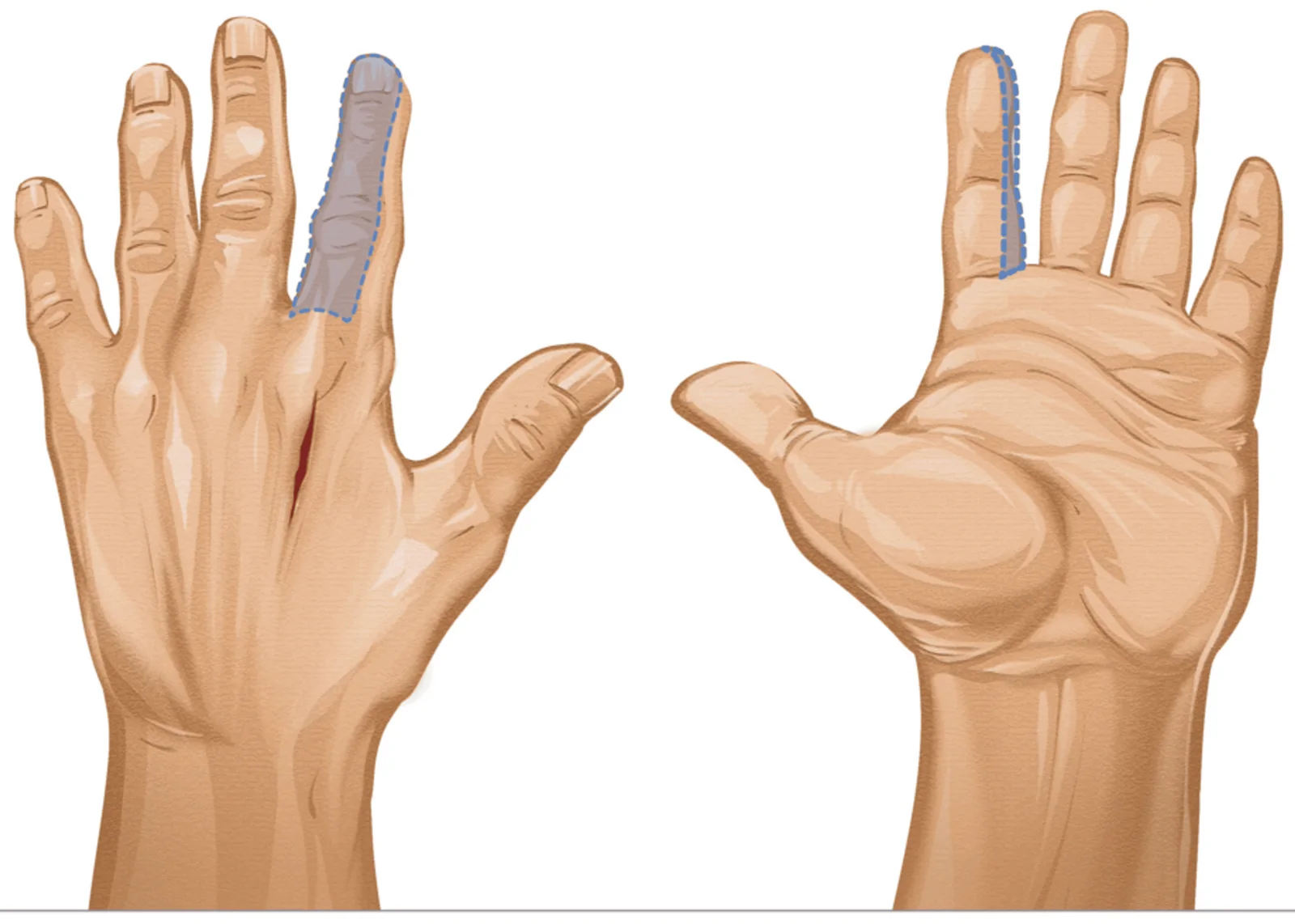
Sensory deficit map of the left hand. Distribution of decreased sensation following injury. Purple shading with dashed borders indicates the patient-reported sensory deficit involving the dorsal and ulnar aspects of the left index finger, with sparing of the true palmar surface. The red mark indicates the location of the dorsal hand laceration. Image created using Adobe Photoshop (Adobe Inc., San Jose, CA).

With gentle traction, the structure appeared tethered distally beneath the skin, with a free proximal end, but it did not result in movement of the digits. Neurovascular examination demonstrated intact distal pulses and preserved motor function. However, a new sensory deficit was noted over the dorsal and medial aspects of the index finger (Figure 2).

The protruding structure was suspected to represent a transected cutaneous sensory nerve, likely a branch of the dorsal digital nerve. The presumed mechanism involved the cat claw dissecting along the nerve proximally, followed by tension, transection far proximal to the skin insult, and partial exteriorization. The exposed structure was clipped, and the wound was irrigated and managed conservatively.

After gaining written consent for the case report, and to confirm the identity/composition of the strand as a nerve fascicle, the threadlike structure was placed into a specimen cup and ordered as a *surgical specimen, foreign body* of low priority to pathology. This specimen, being low priority, was in storage for approximately 1 week, examined only grossly, and described as *fibrous material*, then was to be retained for two weeks and disposed of afterward. However, the medical student went to the pathology lab to inquire (intervening narrowly before disposal of the specimen), and after discussing the sample and case, a frozen section was attempted. Though the tissue had become very dry and damaged, it was at this point positively identified as a nerve fascicle. The amended pathology report: “This case was originally examined and signed out at a ‘gross examination only.’ However, after discussion with the clinical team, a frozen section analysis was completed on the fresh/unfixed tissue and demonstrates findings consistent with a small fragment of neural tissue compatible with nerve.”

A hand surgery specialist was not consulted; this decision was made with informed consent from the patient. The sensory deficit, being not associated with motor dysfunction, was determined not to cause significant disability. Given the small wound with the significant length of the nerve and the age of the patient, it was decided that attempting nerve anastomosis would require a larger wound/incision and would not have a benefit that outweighed the increased risk of infection. The patient was counseled to return if signs of infection presented themselves; she had a persistent sensory deficit for which the medical team was able to objectively account by identifying and differentiating the nerve tissue. The patient had not followed up as of the publication of this paper.

## Discussion

This case represents a rare and unusual presentation of peripheral nerve injury involving exteriorization of a nerve fascicle following minor penetrating trauma. The dorsal digital nerves, which provide sensory innervation to the dorsum of the fingers, are relatively superficial and susceptible to injury [4,5].

The mechanism of injury in this case likely involved penetration by a cat claw followed by dissection along the path of least resistance, including the neurovascular plane. Traction forces may have contributed to partial avulsion and nerve externalization. The patient’s localized sensory deficit supports involvement of a dorsal digital nerve branch. A possible alternative mechanism is traction by the patient herself, though she stated it was painful to the touch.

To our knowledge, exteriorization of a peripheral digital nerve fascicle following a low-energy cat scratch injury has not been previously reported in the literature, highlighting the unusual mechanism and clinical presentation of this case.

Unusual peripheral nerve injuries have been described in the setting of minor trauma, traction mechanisms, and animal-related injuries. Digital nerve injuries may occur following seemingly trivial penetrating trauma, and animal bites have been associated with deeper structural injury than initially apparent [6,7]. Additionally, nerve tissue may be misidentified as foreign material or encountered unexpectedly during minor procedures, emphasizing the importance of anatomical recognition in atypical presentations. However, none of these reports describe true exteriorization of a nerve fascicle through the skin as observed in this case.

In communication with pathology, the initial specimen submission lacked sufficient clinical detail, resulting in limited evaluation. Only after direct clarification was histologic confirmation obtained. Proper specimen handling, including placement in formalin, would have improved tissue preservation and diagnostic quality [8]. Additionally, specimens labeled nonspecifically may not undergo detailed analysis unless explicitly requested. Clinicians should provide clear clinical context and diagnostic intent when submitting specimens to ensure appropriate processing.

The limitations of this analysis include the subjectivity of the story from the patient, the use of frozen section analysis on a dry and damaged specimen, and the lack of follow-up on the patient. These are common limitations in evaluating patients in the emergency room, or in this case, the triage area, after which the patient departed. It must be stated that it is only a presumption that the tissue was a digital nerve fascicle; the evidence to suggest so is the location of the injury, the sensory distribution, the *fibrous material* description of the original gross pathology evaluation (to presume the specimen to be a fascicle), and the later *compatible with nerve* finding in the final pathology report.

From a clinical perspective, this case highlights the importance of recognizing anatomical structures in atypical presentations. A threadlike structure emerging from a wound may be misidentified as a foreign body, suture material, or debris. Failure to recognize neural tissue may result in suboptimal management, inappropriate damage from attempting to remove native tissue, or missed opportunities for appropriate documentation and follow-up. Clinicians should maintain a high index of suspicion for underlying anatomical structures when evaluating atypical wound findings, as even minor trauma may result in unexpected nerve injury.

## Conclusions

This case illustrates an unusual presentation of peripheral nerve injury involving exteriorization of a peripheral nerve consistent with a nerve fascicle following a cat scratch. It emphasizes the importance of anatomical recognition, thorough clinical assessment, and clear interdisciplinary communication. Accurate specimen labeling and appropriate preservation are essential to optimize diagnostic outcomes. Even minor injuries may yield unexpected findings with significant educational value. This case serves as a reminder to be prepared with appropriate consent, a tentative plan, and an understanding of specimen handling protocols when clinically appropriate.
